# A Case Illustrating Challenges: Early‐Onset Signet Ring Cell Rectal Carcinoma With Fulminant Peritoneal Progression

**DOI:** 10.1002/ccr3.72322

**Published:** 2026-03-17

**Authors:** Shivraj Sharma, Madhav Raj Karki, Aron Neupane, Shristi Gupta, Dikshya Pokhrel, Kshitiz Parajuli, Nikhil Agarwal

**Affiliations:** ^1^ Department of Surgical Oncology Birat Medical College Teaching Hospital Morang Nepal

**Keywords:** neoadjuvant chemoradiotherapy, peritoneal carcinomatosis, rectal carcinoma, signet ring cell carcinoma, young adult

## Abstract

Signet ring cell carcinoma (SRCC) of the rectum is a rare histologic subtype and is often associated with aggressive behavior and poor outcomes, particularly in young patients. We report a 19‐year‐old male who presented with acute intestinal obstruction and was diagnosed with rectal SRCC, radiologically staged as cT2N1bM0, acknowledging the limitations of radiologic staging in this histologic subtype. An emergency diversion colostomy was performed, followed by long‐course neoadjuvant chemoradiotherapy (nCRT). Three months later, the patient presented again with abdominal pain and cachexia. Radiology showed evidence of extensive peritoneal disease. Exploratory surgery revealed diffuse peritoneal carcinomatosis, preventing the planned complete cytoreduction and hyperthermic intraperitoneal chemotherapy. Further treatment options were discarded by the family. The disease progressed rapidly, and the patient passed away one month later. This case highlights the challenges in early‐onset rectal SRCC. The aggressive clinical course even after long‐course neoadjuvant chemoradiotherapy, the limitations of current treatment protocols, and the high risk of understaging through conventional radiology are highlighted in this case report.

## Key Clinical Message

Rectal signet ring cell carcinoma in young patients is highly aggressive and prone to radiologic understaging. Despite localized imaging findings, clinicians should maintain high suspicion for rapid peritoneal dissemination.

## 1 Introduction

Colorectal carcinoma (CRC) remains a major global health concern and is increasingly reported in younger populations. In Nepal, CRC accounted for approximately 4.6% of new cancer cases over the past five years [1]. Signet ring cell carcinoma (SRCC) represents a rare pathological subtype, accounting for less than 1% of colorectal cancers and approximately 1%–2% of rectal cancers, and is associated with aggressive behavior and a poor prognosis [2]. Early detection and a multidisciplinary, individualized approach to management are essential, as delayed diagnosis often results in advanced disease at presentation and significant therapeutic challenges in management.

This case is notable for early‐onset rectal SRCC presenting with acute obstruction, followed by rapid peritoneal dissemination despite standard neoadjuvant chemoradiotherapy (nCRT). The transition from apparently localized disease to diffuse carcinomatosis illustrates the biological aggressiveness of SRCC and the limitations of conventional staging modalities in this histologic subtype.

## 2 Case Presentation

### 2.1 Clinical Timeline Summary

• Week 0: Presentation with obstruction → emergency diversion colostomy.

• Week 1: MRI pelvis and biopsy → cT2N1bM0 rectal SRCC.

• Weeks 2–14: Long‐course nCRT (capecitabine‐based).

• Approximately 12 weeks after completion of nCRT: Re‐presentation with abdominal pain, cachexia.

• Exploration: Diffuse peritoneal carcinomatosis → unresectable disease.

• Death: Approximately 4 months from initial diagnosis/one month after exploration.

### 2.2 Case History/Examination

A 19‐year‐old male presented with three days of abdominal distension and obstipation, followed by colicky abdominal pain and vomiting. He reported a six‐week history of altered bowel habits and intermittent fresh rectal bleeding. There was no significant past medical or family history.

On examination, the patient was tachycardic but hemodynamically stable, with a body mass index of 21.58 kg/m^2^. Abdominal distension with generalized tenderness and absent bowel sounds was noted. Digital rectal examination revealed a circumferential mass located 5–6 cm from the anal verge.

### 2.3 Differential Diagnosis, Investigations and Treatment

Laboratory investigations demonstrated mild anemia (hemoglobin 10.6 g/dL), thrombocytosis (511,000/μL), elevated transaminases (approximately three‐fold increase), low total protein (4.6 g/dL), and mildly elevated carcinoembryonic antigen (CEA 6.1 ng/mL; reference < 5 ng/mL).

**FIGURE 1:**
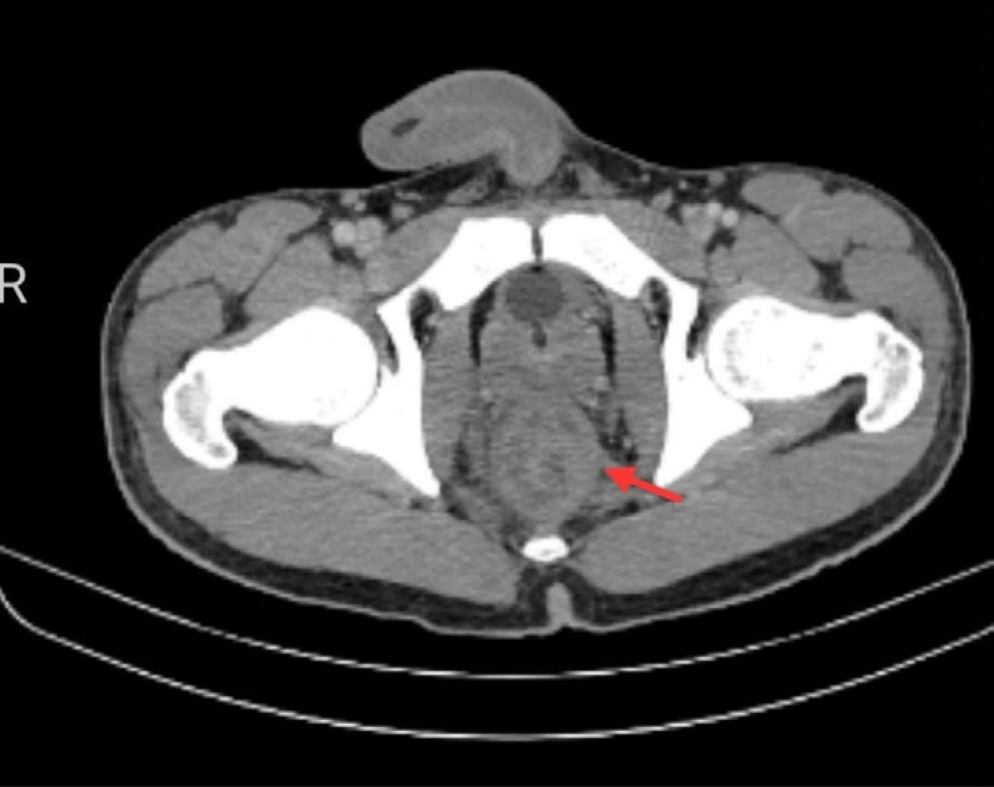
CECT abdomen demonstrated circumferential anorectal wall thickening measuring approximately 18 mm with luminal narrowing.

Contrast‐enhanced computed tomography of the abdomen revealed circumferential anorectal wall thickening measuring approximately 18 mm with luminal narrowing, associated perirectal fat stranding, multiple mesorectal lymph nodes (4–5), and upstream bowel dilatation consistent with acute intestinal obstruction (Figure 1). An emergency laparoscopic diversion loop colostomy was therefore performed.

Postoperatively, a rectal punch biopsy and contrast‐enhanced magnetic resonance imaging (MRI) of the pelvis were performed. MRI demonstrated asymmetric thickening involving the upper, mid, and lower rectum with irregular heterogeneous signal intensity in the mesorectal fat and nodal involvement, consistent with cT2N1b disease, with no radiologic evidence of distant metastasis.

**FIGURE 2:**
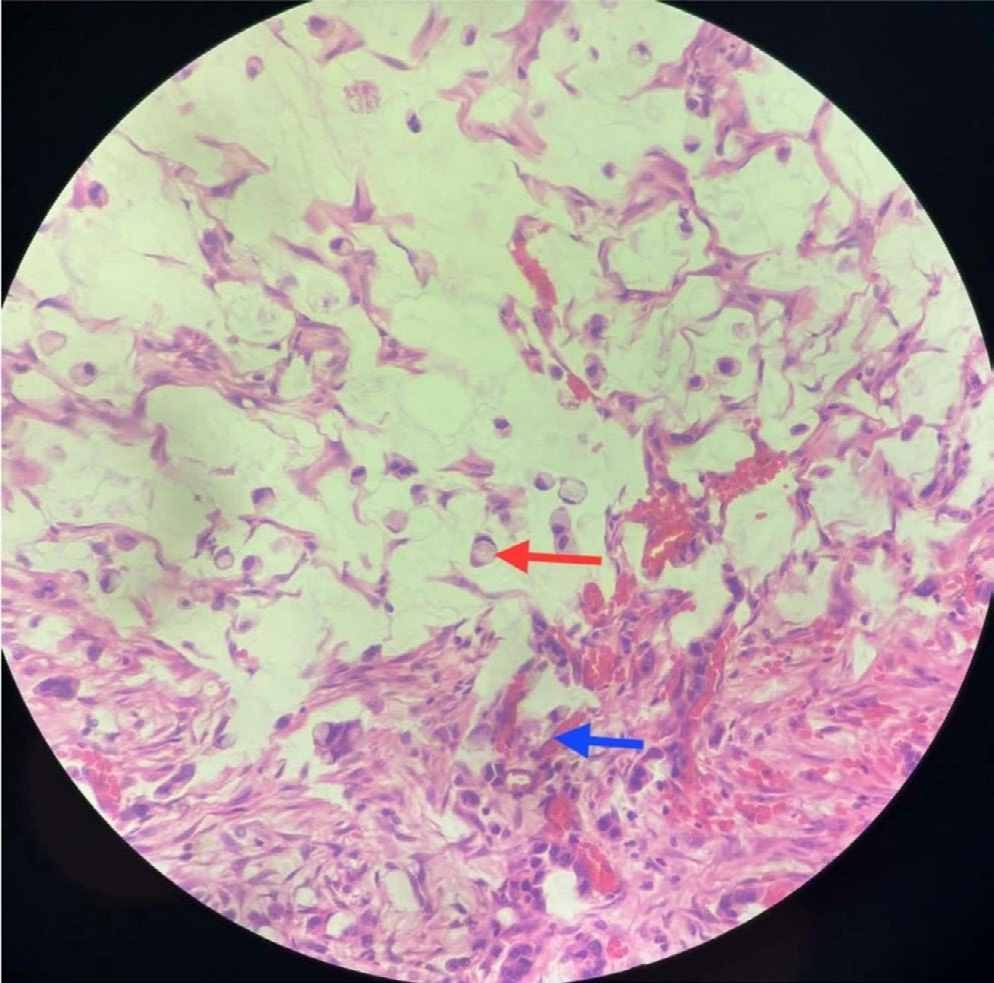
Histopathological slide showing adenocarcinoma deposit having signet ring cell morphology (red arrow) along with lymphatic and/or vascular emboli present (blue arrow).

Histopathological examination revealed adenocarcinoma with extensive signet ring cell morphology and lymphovascular emboli. Although precise quantification was not available, signet ring cells constituted the majority of the tumor, fulfilling diagnostic criteria for SRCC and suggesting poorly cohesive tumor biology (Figure 2). Immunohistochemistry showed tumor cells positive for cytokeratin (AE1/AE3) and positive periodic acid–Schiff staining. Molecular profiling, including mismatch repair or microsatellite instability status, was not performed due to resource limitations.

Following a multidisciplinary discussion, long‐course nCRT with concurrent capecitabine was initiated, considering the obstructive presentation, nodal involvement, circumferential disease, and aggressive histology, despite the apparent early T stage. The patient was discharged with the stoma in situ on oral antibiotics and analgesics.

## 3 Conclusion and Results

Three months later, the patient presented again with severe abdominal pain and weight loss. Exploratory laparotomy revealed extensive peritoneal disease, including omental caking, mesenteric nodules, serosal deposits, and ascites, consistent with diffuse peritoneal carcinomatosis. Biopsy specimens from omental and mesenteric nodules, including three lymph nodes sampled intraoperatively, were all positive for metastatic adenocarcinoma.

Given the extensive disease burden, curative resection and complete cytoreduction were not feasible. Further radical surgery was declined by the family following intraoperative counseling, and the procedure was aborted after the placement of an intra‐abdominal drain. The patient required intraoperative transfusion of two units of packed red blood cells.

On postoperative day 2, the patient developed tachypnea and fever. On postoperative day 4, he developed paralytic ileus, which was managed conservatively. The intra‐abdominal drain was removed on postoperative day 16, and the patient was discharged in stable condition on postoperative day 17. Despite supportive and palliative care, his condition deteriorated, and he died one month later.

## 4 Discussion

Although CRC has been considered a disease of older adults, its incidence among adolescents and young adults is increasing worldwide, including in South Asian countries [1, 3]. Within this spectrum of CRC, SRCC represents a rare (1%–2% of rectal cancers) and aggressive histologic subtype [2, 4, 5].

SRCC is characterized by abundant intracellular mucin displacing the nucleus peripherally, producing the classical signet ring appearance [5, 6]. Clinically, SRCC is associated with younger age, lymphovascular invasion, nodal involvement, and a marked tendency for peritoneal dissemination rather than hematogenous spread [5, 7].

The hallmark of this case report is the rapid progression from radiologically staged cT2N1bM0 disease to diffuse peritoneal carcinomatosis within a short interval. This course strongly suggests radiologic understaging at presentation, likely reflecting the limitations of cross‐sectional imaging in detecting microscopic peritoneal disease, particularly in poorly cohesive histologies such as SRCC. Although diagnostic laparoscopy may improve staging accuracy, it was not feasible in our emergency setting.

Despite radiologic staging suggesting early T‐stage disease, the presence of nodal involvement, circumferential tumor, obstructive presentation, and signet ring cell histology influenced the multidisciplinary decision to proceed with long‐course nCRT.

Emerging literature suggests that SRCC is biologically heterogeneous. Studies, primarily from gastric cancer cohorts, indicate that increasing proportions of signet ring cells are associated with more aggressive behavior, poorer prognosis, and variable treatment response [6, 8]. While extrapolative, this framework offers a potential explanation for the discordance between the patient's early radiologic staging and the subsequent aggressive dissemination.

Young‐onset CRC has also been linked to a higher propensity for peritoneal metastasis and poorer outcomes [5]. Molecular and genetic differences in tumors arising in adolescents and young adults have been reported, further supporting biological distinctiveness [4]. The lack of molecular profiling in this case represents a limitation but reflects real‐world constraints in resource‐limited settings.

Therapeutically, emergency diversion was required to relieve obstruction and stabilize the patient. Long‐course nCRT was administered in accordance with established management strategies for locally advanced rectal cancer. Following the discovery of peritoneal disease, cytoreductive surgery with HIPEC was considered; however, the inability to achieve complete cytoreduction shifted management from curative to palliative intent [7, 9, 10].

Peritoneal carcinomatosis from colorectal cancer carries a poor prognosis, particularly in SRCC histology [11, 12]. Survival outcomes for colorectal SRCC remain dismal compared to purely adenocarcinoma, showing limited benefit from aggressive surgical approaches when peritoneal dissemination is detected [13, 14].

## 5 Conclusion

Despite seemingly localized radiologic staging and adherence to guideline‐based therapy, early‐onset rectal SRCC may have an aggressive course with rapid peritoneal spread. In poorly coherent histologies, this case demonstrates the shortcomings of staging techniques and highlights a poor prognosis once peritoneal dissemination occurs.

## Author Contributions

**Shivraj Sharma:** conceptualization, project administration, resources, supervision, validation. **Madhav Raj Karki:** writing – original draft, writing – review and editing. **Aron Neupane:** writing – original draft, writing – review and editing. **Shristi Gupta:** project administration, resources, writing – original draft. **Dikshya Pokhrel:** resources, writing – original draft. **Kshitiz Parajuli:** resources, writing – original draft. **Nikhil Agarwal:** resources, writing – review and editing.

## Funding

The authors have nothing to report.

## Consent

Written informed consent was obtained from the patient's legal next of kin to publish this report in accordance with the journal's patient consent policy.

## Conflicts of Interest

The authors declare no conflicts of interest.

## Data Availability

The data that support the findings of this study are available from the corresponding author upon reasonable request.
